# Corrected and republished from: “Clinical validation of an RSV neutralization assay and analysis of cross-sectional sera associated with 2021–2023 RSV outbreaks to investigate the immunity debt hypothesis”

**DOI:** 10.1128/spectrum.01739-25

**Published:** 2025-12-23

**Authors:** Eli A. Piliper, Jonathan Reed, Alexander L. Greninger

**Affiliations:** 1 Department of Laboratory Medicine and Pathology, University of Washington Medical Center, Seattle, Washington, USA; 2 Vaccine and Infectious Disease Division, Fred Hutchinson Cancer Research Center 7286 , Seattle, Washington, USA; Quest Diagnostics Nichols Institute, Chantilly, Virginia, USA

**Keywords:** RSV, neutralization, titer, epidemiology, seroepidemiology, outbreaks, respiratory syncytial virus, correlate of protection, correlates

## Abstract

Respiratory syncytial virus (RSV) is a leading cause of acute respiratory infections and hospitalization in infants and the elderly. Newly approved vaccines and the prophylactic antibody nirsevimab have heightened interest in RSV immunologic surveillance, necessitating the development of high-throughput assays assessing anti-RSV neutralizing activity. Quantitative viral neutralization remains the best correlate of protection for RSV infection and the gold standard for RSV immunological testing. Here, we developed a high-throughput RSV strain A2 focus-reduction neutralization test validated to CLIA/GCLP standards using both clinical specimens and commercially available reference sera. The assay is highly accurate, generating reference serum neutralizing titers within twofold of established assays, with an analytical measurement range between 8 and 1,798 international units (IU) per mL. Neutralizing activity measured by the assay strongly correlated with antibody titer determined via indirect ELISA (*ρ* = 1.0, *P* = 0.0014). Individuals recently having tested RT-qPCR positive for RSV had a 7.5-fold higher geometric mean neutralizing titer relative to RSV PCR negatives (*P* < 0.0001). The validated assay was then used to investigate the immunity debt hypothesis for resurgent RSV outbreaks in the 2022–2023 season, using adult clinical remnant sera sent for HSV-1/2 antibody testing. There was no difference in geometric mean anti-RSV neutralizing titers between sera sampled before and after the 2022–2023 RSV outbreak (*P* = 0.68). These data are consistent with limited changes in RSV neutralizing antibody levels in adults across the 2022–2023 RSV outbreak.

**IMPORTANCE:**

Population surveillance studies of serum neutralizing activity against RSV are crucial for evaluating RSV vaccine efficacy and vulnerabilities to new strains. Here, we designed and validated a high-throughput assay for assessing anti-RSV neutralizing activity, standardized its measurements for comparison with other methodologies, and demonstrated its applicability to real-world samples. Our assay is precise, linear, and yields measurements consistent with other standardized assays, offering a methodology useful for large-scale studies of RSV immunity. We also find no significant difference in neutralizing titers among adults between taken before and after large RSV outbreaks associated with the latter stages of the COVID-19 public health emergency, underlining the need for a greater understanding of the dynamics of serological responses to RSV infection.

## INTRODUCTION

Respiratory syncytial virus (RSV) is one of the most common causes of lower respiratory tract infections worldwide, infecting approximately 64 million people and causing at least 160,000 deaths and 3 million hospitalizations each year (1, 2). Recent years have seen multiple revolutions in RSV disease prevention, including the FDA approval of three vaccines for older adults and pregnant individuals as well as the prophylactic monoclonal antibody (mAb) nirsevimab for prevention of RSV lower respiratory tract disease in neonates and infants (3–5). The past 2 years have also seen global resurgence of RSV infections, with a recent example being the 2022–2023 outbreak. This resulted in unexpectedly high case counts relative to prior years and contributed to hospital burden during the COVID-19 pandemic. Immunity debt attributed to prolonged lack of exposure to RSV during the COVID-19 pandemic has been suggested as possible cause (3, 6–9), while others postulate that immune changes related to SARS-CoV2 infection or changes in diagnostic practices may also be involved (10–14). These developments necessitate monitoring population immunity and vaccine immunogenicity, both best assessed by measuring neutralizing antibodies against RSV (15–19). RSV neutralizing antibody titer is a major correlate of protection, with higher neutralizing titers being associated with lower viral load and reduced risk of severe RSV-induced disease (20–25).

The standard for neutralization assays, the plaque-reduction neutralization test (PRNT), requires 3–5 days of incubation time and is less amenable to high-throughput applications (18). An alternative is the focus-reduction neutralization test (FRNT), which often requires less sample, reagent volume, and incubation time (26–30 h) to measure neutralizing titers. The RSV FRNT assay can be performed in a 96-well plate format portable to liquid handlers for higher throughput and less inter- and intra-assay variability (26–29). These assays are typically performed using the Hep-2 or VeroE6 cell lines, both of which are established in RSV culture. Challenge viruses used are generally research strains, primarily RSV A2 and A Long (30). Strain-specific reporter viruses are powerful for high-throughput applications but require the development of a recombinant virus, which may be more challenging to update for future strains (31). An FRNT using chromogen-conjugated secondary antibodies to visualize foci is easy to implement and scalable to high-throughput applications such as the serological response to vaccination and population immunity monitoring. In addition, the assay can be adapted to other culturable RSV isolates to allow for evaluation of emerging vaccine or therapeutic resistance (32).

Here, we describe validation of a RSV FRNT, an automation-ready, high-throughput, standardized method for assessing serum neutralizing activity against RSV in clinical, clinical trial, and research settings. In addition, we use the RSV FRNT to monitor population immunity against RSV in remnant serum specimens taken before, during, and after the 2022-2023 RSV outbreak.

## RESULTS

### Standardization and accuracy

To evaluate accuracy, we compared neutralizing titers of commonly used reference sera measured by our RSV FRNT assay against previously published results (33, 34). Since neutralizing assays between labs can produce varied neutralizing titers even when testing the same specimen, we normalized our results to international units per mL (IU/mL) using the First International Standard for Antiserum to Respiratory Syncytial Virus (NIBSC code: 16/284) (35). To convert ND50 neutralization results from the RSV FRNT assay to IU/mL, a mean ND50 neutralizing titer estimate was obtained for NIBSC-16/284 by repeat testing over 3–6 assay runs and the conversion factor determined by taking the ratio of the concentration of the standard in IU/mL over the mean ND50 neutralizing titer result. The conversion factors for RSV A2 and RSV B WV/14617/85 FRNT ND50 units to IU/mL were 0.37 and 0.94, while the ND80 conversion factors were 1.32 and 3.40, respectively (Table S1). RSV A2 was used for all other validation/experiments unless otherwise specified. Next, RSV reference sera were tested in duplicate over 3–6 days by one operator and the mean ND50 neutralizing titer results converted to IU/mL for both RSV A2 (Table 1) and RSV B (Table 2). Our results were within twofold of previously published values for the tested reference sera for both RSV A2 and RSV B (33, 34).

**TABLE 1:** Comparison of neutralization values obtained by the RSV FRNT assay using RSV A2 as the challenge virus with published estimates

Sample	*n*	IU/mL RSV FRNT^*a*^^,*c*^	IU/mL reference^*b*^^,*c*^	Ratio of IU/mL RSV FRNT/reference
NR-21973	5	9,539	6,830	1.4
NR-4020	5	1,392	1,236	1.1
NR-4021	6	4,154	4,404	0.9
NR-4022	6	898	706	1.3
NR-4023	12	1,114	625	1.8

^*a*^ NIBSC 16/284 standard was tested 12 times with RSV A2, with geometric mean 5,404.8. IU/mL was obtained by multiplying ND50 values obtained in the RSV FRNT by the conversion factor 0.37.

^*b*^ Reference values obtained from Crank et al., which used an IU/mL conversion factor of 0.88 (33).

^*c*^ Data represent the geometric mean.

**TABLE 2:** Comparison of neutralization values obtained by the RSV FRNT assay using RSV B WV/14617/85 as the challenge virus with published estimates

Sample	*n*	IU/mL RSV FRNT^*a*^^,*c*^	IU/mL reference^*b*^^,*c*^	Ratio of IU/mL RSV FRNT/reference
NR-21973	6	13,825	12,059	1.1
NR-4022	6	1,137	1,005	1.1

^*a*^ NIBSC 16/284 standard was tested six times resulting in a geometric mean ND50 of 2,127. IU/mL values were obtained by multiplying ND50 measurements by the conversion factor 0.94. McDonald et al. reported a mean conversion factor across all assays of 1.1 but did not report a per-assay conversion factor (34).

^*b*^ Reference values obtained from McDonald et al. (34).

^*c*^ Data represent the geometric mean titer.

**Fig 1:**
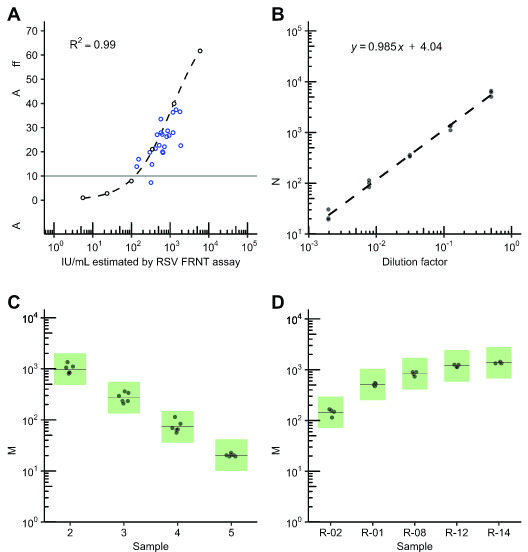
Accuracy, linearity, and precision of an RSV FRNT assay. (**A**) Results of RSV-binding antibody measurement by indirect ELISA of the linearity panel specimens (black open circles; see Assay Accuracy) and the rubella serology remnant specimens (blue open circles) are shown. The dashed black line is the 4-parameter logistic curve fit of the ELISA results from the linearity panel. Sera was measured in duplicate, normalized relative to the assay cutoff control (relative absorbance of 10), and plotted as average values. The standard deviations of the measurements are plotted using black line range though these line ranges are small enough to be obscured by the larger diameter of the plotted points. The gray line depicts the approximate normalized ELISA cutoff value of approximately 10, with relative absorbance between 9 and 11 considered inconclusive. (**B**) Results of triplicate testing of the linearity panel, composed of a serial dilution series of reference serum (NR-21973), was plotted against the expected values for the series ranging from 6 to 5,543 IU/mL. The dashed line is the best fit from the linear regression analysis. The formula for this linear regression is displayed in the upper-right corner. (**C**) Precision testing results of select linearity panel specimens (2 through 5) that were within the expected analytical measurement range of the assay (expected values ranging from 22 to 1,386 IU/mL) were measured in duplicate over 3 days. Green boxes demarcate boundaries of expected variation for a GCV of 37%. Solid lines represent mean IU/mL measurement for panel member. (**D**) Precision testing of five serum specimens from rubella remnant testing spanning the AMR of the assay, tested in duplicate over 2 days. Green boxes demarcate boundaries of expected variation for a GCV of 37%. Solid lines represent mean IU/mL values for panel member.

We next tested if RSV FRNT results were proportional to binding antibody titers in clinical remnant and contrived serum samples, with the expectation that specimens with higher IU/mL values would have higher anti-RSV antibody titers by indirect ELISA (36). We prepared a linearity panel of six contrived specimens derived from a reference serum (NR-21973) with theoretical values ranging from 6 to 5,543 IU/mL. We tested these alongside 22 remnant sera that were seropositive for anti-rubella IgG. The remnant rubella specimen population was 91% female with a median age of 32 years (range 21–42 years). The neutralizing titer of the linearity panel memberwasre positively correlated with the level of RSV-reactive antibodies detected by ELISA (*ρ* = 1, *P* = 0.0014; Fig. 1A). All 22 rubella serology specimens tested positive for neutralizing antibodies by RSV FRNT, with results spanning the full range of measurable IU/mL values without specimen pre-dilution (8–1,798 IU/mL). Similarly, 21 of 22 rubella serology specimens were positive for RSV-reactive antibodies by ELISA based on the manufacturer’s cutoff. The one remnant rubella serology specimen that fell just below the ELISA positive cutoff had a neutralizing titer of 320 IU/mL. The Ig-depleted control yielded a neutralizing titer of <8 IU/mL (below the lowest dilution) in the RSV FRNT assay and fell below the ELISA positive cutoff (data not shown). No correlation was seen between rubella IgG and RSV FRNT levels.

### Linearity

To confirm our measured RSV FRNT titers scale proportionally with expected neutralizing titer, we next analyzed IU/mL results from the linearity panel (described above) by linear regression. Linearity panel IU/mL values, converted from ND50 measurements generated with RSV A2, within or near the assay lowest and highest dilutions (20- to 4,860-fold dilution, or 8 to 1,798 IU/mL) were strongly linear (*R*^2^ > 0.9), with a slope of 0.99 (Fig. 1B; Table S2). This met our assay acceptance criteria of *R*^2^ > 0.9 and slope between 0.9 and 1.1. The linearity panel IU/mL values converted from ND80 measurements were likewise strongly linear (Table S2).

### Precision

Next, we sought to estimate the intra-, inter-, and within-lab imprecision of the RSV FRNT measurements in terms of the coefficient of variation (CV), or the standard deviation divided by the mean. This metric is generally used for quantifying assay variation and, by normalizing for the mean measurement, enables the comparison of variability of a wide range of measurement values (37). Given the log-normal distribution of neutralization data similar published neutralization assays utilize the geometric coefficient of variation (GCV) as an estimate of imprecision rather than the standard CV. Given the more variable nature of cell-based assays, the published acceptable GCV range is broad, ranging from 20% to 50% (38–48). Based on our experiences with neutralizing assay imprecision utilizing cell-based assays, we set an imprecision criterion of a GCV of 37% (geometric standard deviation of 1.43) to ensure that the true population mean is at least within twofold of the sample mean 95% of the time.

To measure imprecision, we ran four linearity panel specimens in duplicate over 3 days (Fig. 1C) and five remnant rubella serology specimens in duplicate over 2 days (Fig. 1D). All assays were performed by one operator. Imprecision of IU/mL values calculated from either ND50 or ND80 measurements were estimated via ANOVA analysis. No specimen, clinical or contrived, exhibited intra-assay, inter-assay, or within-lab GCV above 37% (Tables 3 and 4). Linearity panel specimen within-lab GCV values ranged from 5.5% to 27.8% and 18.7% to 24.9% for ND50 and ND80 measurements, respectively (Fig. 1C, Table 3). Remnant rubella specimens had within-lab GCV values ranging from 3.2% to 15.5% and 3.2% to 17.6% for ND50 and ND80 measurements, respectively (Fig. 1D, Table 4). We also estimated imprecision of RSV FRNT measurements utilizing RSV B WV/14617/85 as the challenge virus and showed that within-lab imprecision for this assay met our criteria of GCV <37% (Table 5).

**TABLE 3:** Intrassay, interassay, and within-lab imprecision of ND50 and ND80 measurements of linearity panel specimens^*b*^

	Panel member	Expected measurement (IU/mL)	*n* ^*a*^	Geometric mean (IU/mL)	Intraassay component		Interassay component		Within-lab	
					Geometric SD	% GCV	Geometric SD	% GCV	Geometric SD	% GCV
ND50	2	1,386	6	983	1.14	12.9	1.20	18.0	1.25	22.3
	3	346	6	272	1.11	10.9	1.24	21.8	1.27	24.5
	4	87	6	73	1.16	14.6	1.26	23.5	1.31	27.8
	5	22	6	20	1.07	6.9	0.00	0.0	1.06	5.5
ND80	2	1,263	6	1,108	1.12	10.9	1.25	22.2	1.28	24.9
	3	315	6	289	1.09	8.9	1.21	19.5	1.24	21.5
	4	79	6	75	1.09	8.7	1.18	16.4	1.20	18.7
	5	20	6	19	1.28	25.2	0.00	0.0	1.25	22.8

^*a*^ Total number of replicates tested.

^*b*^ All neutralizing titer values were averaged. Geometric SD and CV were calculated. Negative variance estimates from ANOVA analysis were reported as a GSD of zero.

**TABLE 4:** Intrassay, interassay, and within-lab imprecision of ND50 and ND80 measurements of remnant clinical specimens^*b*^

Panel member	*n* ^*a*^	IU/mL from ND50			IU/mL from ND80		
		Geometric mean	Geometric SD	% GCV (within lab)	Geometric mean	Geometric SD	% GCV (within lab)
R-01	4	510	1.06	6.0	600	1.06	6.0
R-02	4	145	1.17	15.5	143	1.19	17.6
R-08	4	835	1.09	8.7	983	1.09	8.7
R-12	4	1,190	1.05	5.1	1,345	1.10	9.3
R-14	4	1,367	1.03	3.2	1,609	1.03	3.2

^*a*^ Total number of replicates tested.

^*b*^ All neutralizing titer values were averaged, and geometric SD and total within-lab imprecision was estimated.

**TABLE 5:** Intrassay, interassay, and within-lab imprecision of ND50 and ND80 measurements of contrived specimens using RSV B WV/14617/85 as challenge virus^*b*^

Sample	*n* ^*a*^	Geometric mean (IU/mL)	Intraassay component		Interassay component		Within-lab	
			Geometric SD	% GCV	Geometric SD	% GCV	Geometric SD	% GCV
NIBSC 16/284	6	2,000	1.28	24.8	1.11	10.6	1.31	27.1
NR-21973	6	13,825	1.26	23.5	1.02	2.1	1.26	23.6
NR-4022	6	1,137	1.05	5.2	1.11	10.5	1.12	11.8

^*a*^ Total number of replicates tested.

^*b*^ Three reference sera were tested in duplicate over 3 days with RSV B WV/14617/85 in place of RSV A2. All neutralizing titer values were averaged. Geometric SD and GCV were calculated.

### Assay analytical measurement range, limit of detection, and clinical reportable range

Next, we determined the lowest and highest RSV FRNT measurements that met both our imprecision and linearity criteria. Based on the imprecision analysis of the linearity panel and remnant rubella sera, the within-lab GCV calculated from either ND50 or ND80 was less than 37% at the minimum and maximum dilutions of the assay (20- and 4,860-fold, Tables 3–5). Thus, the lower limit of quantitation and upper limit of quantitation of the assay were determined to be 20 and 4,860, respectively, for both ND50 and ND80. This range corresponded to 8–1,798 IU/mL using the RSV A2 conversion factor for ND50. Because measurements through this range were also linear (Table S2), this range also defines the analytical measurement range (AMR), or the range of neutralizing titers that meet both linearity and precision requirements.

The limit of detection (LoD) of the assay is the minimum percent inhibition required that can be distinguished from an uninhibited control (49) and thus establishes negative cutoff for assay signal in the form of percent inhibition. First, the limit of blank was determined to be 14.5% inhibition (Table S3) based on the mean percent inhibition and standard deviation of negative control wells receiving virus with no serum, which were 0.0% and 8.8%, respectively (Table S4). The LoD was determined to be 28.2% inhibition (Table S3) based on the variation of NR-4023 diluted down to near-LoB level, which was 8.3% (Table S4). Since 50% and 80% inhibition are well above the LoD of the inhibition assay, ND50 and ND80 measurements within AMR are distinguishable from blank.

Since we anticipate serum specimens may exhibit neutralizing titers that exceed the assay AMR, we next wanted to determine if sera could be pre-diluted prior to testing to extend range of reportable values, also known as the clinical reportable range. This was evaluated by preparing four pre-dilutions of NR-21973 to have expected values of 1,386, 346, 87, or 22 IU/mL and running them in triplicate. When accounting for pre-dilution, all neutralizing titer results were within 1.2-fold of the original value of 11,086 IU/mL (Table S5).

### Comparison of 2022–2023 RSV neutralization titers during a period of RSV outbreaks

**Fig 2:**
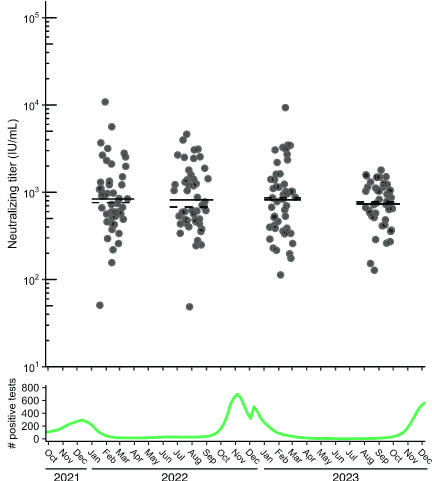
Distribution of neutralizing titers among random population samples collected from late 2021 to early 2024. (Top panel) Neutralizing titer results of HSV serology remnant serum specimens from individuals sampled around February 2022, August 2022, February 2023, and September 2023, with date of serum collection on *x*-axis. For each cross-sectional group, dashed and solid lines represent geometric median and geometric mean, respectively. (Bottom panel) Number of positive RSV antigen tests across time in Washington State taken from CDC Washington State trends (50), reported as 5 week average. Log_2_-transformed data were analyzed, using a two-sided student’s *t*-test to compare groups pairwise.

**Fig 3:**
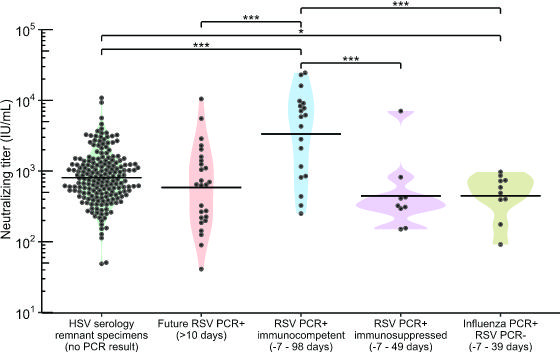
Distribution of neutralizing titers among RSV negative, positive, and future-infected individuals relative to a random population sample. 50% neutralizing titer in IU/mL for individuals sampled from October 2021 to February 2024. Solid lines represent geometric mean. HSV serology remnant specimens: a random population sample of remnant sera sent for clinical HSV remnant testing, collected from February 2021 to September 2023, with no PCR data available for RSV. Future RSV-infected: HSV remnant samples (not part of the HSV serology remnant specimen group above) from individuals who tested PCR positive for RSV 25 or more days after serum collection date. RSV PCR+, immunocompetent: emergency department (ED) patients PCR-positive for RSV 98 days before to 7 days after serum collection. RSV PCR+, immunosuppressed: ED patients PCR-positive for RSV 49 days before to 7 days after serum collection, with medical status of immunosuppression at time of test/collection. Influenza+, RSV−: ED patients with no recorded RSV-positive or immunosuppression status who tested PCR-positive for influenza virus 39 days before to 7 days after serum collection. Log_2_-transformed data were analyzed, using a two-sided student’s *t*-test to compare groups pairwise. *P < 0.05, ***P < 0.001.

We next sought to apply the assay to a relevant clinical research question utilizing clinical specimens. Specifically, we investigate the role of population-level RSV immunity debt, described as a decline in immunity following reduced exposure to RSV (71), RSV outbreak. We determined the geometric mean titer (GMT, geometric mean of IU/mL converted from ND50 measurements) of a cross-sectional collection of 193 remnant sera sent for clinical HSV western blot testing sampled across four discrete timepoints between 2022 and 2023: after the 2021–2022 RSV outbreak (February 2022), before (August 2022) and after (February 2023) the 2022–2023 RSV-outbreak, and before the 2023–2024 RSV outbreak (September 2023, Fig. 2). We found no significant difference in GMT or geometric median titer between any combination of the four timepoints (one-way ANOVA, *P* = 0.86). Moreover, samples collected before and after the 2022–2023 RSV outbreak exhibited no significant difference in GMT (Wilcoxon ranked-sum test, *P* = 0.68, Fig. 2). To further explore whether adults testing positive for RSV in the future were more likely to have lower RSV neutralizing titers, we tested RSV neutralizing titers of remnant HSV serology serum specimens from 26 individuals who tested PCR positive for RSV after serum collection (by 25–463 days), referred to as future-RSV-PCR-positives. The overall GMT from these individuals was not significantly different from the cross-sectional sera for which RSV PCR testing data were not available (two-sided *t*-test, *P* = 0.24, Fig. 3).

To further profile neutralizing titers in individuals testing PCR positive or negative for RSV, we collected remnant sera from UW Medicine patients who had recently (within 3 months of sampling) tested PCR positive for RSV (*n* = 29 individuals) or PCR negative for RSV and positive for influenza virus (*n* = 10 individuals) during the 2023–2024 respiratory season (Fig. 3). The PCR positive for RSV group included 9 immunosuppressed individuals based on chart review and were separated into their own group for this analysis. Neutralizing GMT of the combined cross-sectional HSV serology remnant specimen groups described above was 4.2-fold lower than immunocompetent RSV PCR positive individuals (two-sided *t*-test, *P* = 0.0004), 1.8-fold higher than that of RSV PCR positive immunosuppressed individuals (*P* = 0.17), and 1.8-fold higher than that of influenza PCR-positive, RSV PCR-negative individuals (*P* = 0.04). The future-RSV-PCR-positive samples had a GMT 5.7-fold lower than PCR positive individuals without immunosuppression (*P* = 0.00017) and 1.3-fold higher than PCR positive, immunosuppressed individuals (*P* = 0.56). Immunocompetent RSV PCR positive individuals had a GMT 7.5-fold higher than that of RSV PCR positive immunosuppressed individuals (*P* = 0.0008), and 7.5-fold higher than influenza virus PCR-positive, RSV PCR-negative individuals (*P* < 0.0001). Thus, the measurements obtained by RSV FRNT align with patient medical background and can represent individuals ranging from the recently infected and immunocompetent to the immunosuppressed.

## DISCUSSION

With the recent development and rollout of RSV vaccines and the prophylactic mAb nirsevimab, there is increasing interest in RSV immunosurveillance and understanding RSV immunity dynamics. While ELISAs have proven invaluable for rapid, high-throughput detection of antibodies in diagnostic testing and longitudinal studies (51, 52), neutralization assays offer a means of functionally assessing antibodies against a given RSV strain (53). This functionality is needed when considering the poor immunogenicity of previous vaccine candidates (54), immune evasion techniques employed by RSV (55), and the continued correlation between neutralizing RSV antibodies and protection against infection and hospitalization (20–25). The PRNT assay remains the gold standard (56) for measuring neutralizing antibodies but is limited in throughput and speed (18), both of which are improved in neutralization assays measuring focus reduction.

Here, we designed and validated a high-throughput RSV neutralization assay amenable to clinical testing, clinical trials, and seroprevalence studies. As a precise, sensitive, and linear assay standardizable to the NIBSC 16/284 RSV neutralizing reference material, RSV FRNT is an alternative to reporter virus methods, allowing for similar throughput, quantification, and precision that does not require recombinant viruses. The potential to use other RSV strains (preceded by validation) without genetic modification could prove useful for monitoring for drug resistance and vaccine evasion by RSV evolution, which are potential concerns given the antigenic variability of the RSV F-protein (57–59), continued vaccine roll-out (4), and recent identification of RSV strains resistant to nirsevimab (60, 61).

A variety of assay formats have been developed to measure serum RSV neutralizing activity (62) and our assay had similar if not improved precision compared to other assays (38–43). Neutralizing activity measured with RSV FRNT correlated with RSV antibodies detected by ELISA (Fig. 1A) and was absent in Ig-depleted sera (data not shown), showing that the assay is both specific and sensitive to anti-RSV antibodies. Our RSV FRNT differs from most other assays with a somewhat lower upper AMR limit of only 4,860 ND50 or ND80, corresponding to 1,798 IU/mL or 6,415 IU/mL, respectively (30). Given that only 2.6% (*n* = 6/226) of tested clinical specimens exhibit a neutralizing titer >10,000 ND50 (3,700 IU/mL), we found the increased throughput more desirable. Studies of recently infected or vaccinated individuals may require additional pre-dilution with this assay. We also standardized our RSV FRNT to the First International Standard for Antiserum to Respiratory Syncytial Virus strains A and B, which has been reported for select assays (62). Our RSV A2 conversion factor from ND50 to IU/mL of 0.37 is very similar to the 0.38 conversion factor used in a 2021 RSV vaccine clinical trial (63). Alternative conversion factors (33) are most likely explained by key differences in assay methodology, such as challenge virus, target cell line, and assay readout (30, 62). Our RSV B conversion factor from ND50 to IU/mL was 0.94, similar to the average conversion factor of 1.1 calculated from NIBSC (34).

We found no significant difference in neutralizing titer between individual serums sampled pre- and post-RSV 2022–2023 outbreak, unlike earlier studies which found evidence of reduced anti-RSV neutralizing titer during the COVID-19 pandemic (17, 64). A 2022 population-immunosurveillance study using methods similar to ours also did not detect reduced anti-RSV immunity prior to RSV resurgence (65). Moreover, a recent study attributed approximately two-thirds of the increase in RSV cases to significant increases in RSV testing volumes (14). Other theories behind post-pandemic RSV resurgence, such as SARS-CoV-2-induced immune dysregulation (10–13), changes in population-wide health-seeking behavior (66), and loosening of public health safety measures (9) should also be investigated to inform future vaccination and immunotherapeutic development.

Limitations of this study include its cross-sectional design and convenience sampling of remnant clinical specimens for validation. Measurement of pre-and post-outbreak RSV neutralizing titers were limited by the sample size (*n* = 226). Our sample group lacked infants and had a limited number of elderly individuals (*n* = 22, 12.4%), both of which are especially vulnerable to lowered anti-RSV immunity and severe RSV symptoms (2, 67, 68). Patients with no identified RSV infection might have experienced unreported RSV infection from the smaller outbreak of winter 2021, potentially inflating neutralizing titers and confounding comparison of pre- and post-2022 outbreak populations. RSV strains A2 and B WV/14617/85, classically used for FRNT and PRNT validation (62), were used instead of more contemporary isolates in circulation during the 2022–2023 pandemic (69–71). Differences in specimen handling may have influenced measurements. For example, we noticed that GMT across sera of RSV PCR-positive individuals (without immunosuppression) sourced from one hospital trended slightly lower compared to those from the two other hospitals used in the study by 1.81-fold and 0.89-fold, respectively (Fig. S1) although this difference did not reach statistical significance.

Our findings establish the RSV FRNT assay as a high-throughput, accurate neutralization assay sufficiently sensitive to distinguish immunosuppressed and seroconverted individuals from the general population, a diagnostic necessity given the upcoming release of the RSV vaccine. Our immunosurveillance study using a highly validated assay found no evidence of RSV immunity debt in adults, underlining the challenges of associating RSV epidemiology with population immunity dynamics.

## MATERIALS AND METHODS

### Virus and cell line

Assays were run with RSV A2 as the challenge virus (ATCC, VR-1516), unless otherwise specified. RSV B strain WV/14617/85 (ATCC, VR-1400) challenge experiments are described in Supplemental Methods. VeroE6 cells were seeded in DMEM-10 in transparent 96-well plates (Corning, 3585) at 10,000 cells/well and grown for 24 h prior to infection.

### Growth of challenge virus

RSV strain A2 was grown in Hep-2 cells (ATCC CCL-23), while RSV strain B WV/14617/85 was grown in VeroE6 cells (ATCC, CCL-1587). The following procedure was used for growing both strains: Cells were seeded in T-75 flasks (Corning, 430614U) to reach ~80%–90% confluency overnight. On the day of infection, cells were first washed with DPBS (Gibco, 14190-144). Virus was then resuspended in DMEM-2 (for VeroE6 cells) or MEM-2 (for Hep2 cells). DMEM-2 was made from high-glucose DMEM with glutamax and sodium pyruvate (Thermofisher, 10569010), 10 mM HEPES (Thermofisher, 15630080), 1% penicillin-streptomycin (Thermofisher, 15140122), and 2% heat-inactivated FBS (Thermofisher, A3840001). MEM-2 was made from MEM with L-glutamine (Thermofisher 11095080), 10 mM HEPES (Thermofisher, 15630080), 1% penicillin-streptomycin (Thermofisher, 15140122), and 2% heat-inactivated FBS (Thermofisher, A3840001). Resuspended virus was then added to cells at 0.01 to 0.1 MOI. Flasks were incubated at 37°C 5% CO_2_ until 80% or more of the monolayer was either destroyed or manifesting syncytia (3–7 days). Virus stocks were passaged three times from original ATCC stock (lot #70050739). Virus was then harvested (at P3) by scraping off cells into flask supernatant. This material was centrifuged at 4°C for 5 min at 200 × *g*. Following removal of supernatant from the cell pellet, the pellet was resuspended in media and subjected to three freeze-thaws to release cell-bound virions. Media from the freeze-thawed pellet was combined with original supernatant and mixed thoroughly. This mixture was diluted twofold with sterile-filtered 50% (w/v) sucrose (JT Baker, 4097-04, resuspended in DPBS) to produce virus stock with 25% (w/v) sucrose to reduce degradation of virion infectivity in storage (72). This mixture was aliquoted into 200 µL volumes and stored in −80°C. Aliquots were only freeze-thawed once before being discarded to minimize titer variation from viral degradation. One of these aliquots was subsequently titered by serial dilution via a focus-forming assay (see Focus-forming assay). Based on this titer, working stocks were pre-diluted in media to reach 150–250 foci per well at time of microneutralization assay.

VeroE6 cells (ATCC, CCL-1587) for neutralization assays were maintained below 30 passages and grown in DMEM-10, consisting of high-glucose DMEM with glutamax and sodium pyruvate (Thermofisher, 10569010), 10 mM HEPES (Thermofisher, 15630080), 1% penicillin-streptomycin (Thermofisher, 15140122), and 10% heat-inactivated FBS (Thermofisher, A3840001).

### Construction of contrived specimens

Ig-depleted pooled serum specimens (NR-49447, BEI Resources, and S5393, Sigma-Aldrich) were used as negative controls for assay validation, as both exhibited neutralizing activity below the LoD. Anti-RSV reference serum NR-21973 was obtained from BEI Resources, determined to have an ND50 of 29,963 by the RSV FRNT, was diluted twofold, then serially diluted fourfold with DMEM-10 to produce six contrived specimens along a spectrum of theoretical ND50s (14982, 3745, 936, 234, 59, 15). These contrived specimens were split into single-use aliquots to minimize freeze-thaw effect and stored at −80°C.

### Clinical specimens

Plasma or sera from patients with a history of positive RT-PCR test for RSV or influenza virus since 1 October 2023 were obtained from University of Washington (UW) Medicine patients that were sampled during peak RSV incidence (mid-November 2023 to January 2024) and no later than 40 days following PCR testing (Fig. S1). Remnant serology specimens sent for rubella IgG testing were obtained from UW Virology. Cross-sectional sera were obtained from remnant specimens sent for HSV-1/2 Western Blot testing in February 2022, July 2022, February 2023, and July 2023 (*n* = 47 for each timepoint). Individual line-item data are provided in Table S6.

### Indirect ELISA

Samples were measured with anti-RSV Human IgG ELISA (Abcom ab108765) per manufacturer protocol, using included 96-well plates. Plate absorbance was measured at 450 nm and 620 nm immediately after adding stop solution, and optical density was background-corrected by calculating the difference of 450 nm and 620 nm. Substrate blanks, negative controls, cut-off controls, and positive controls were checked to be within manufacturer criteria. Per manufacturer instructions, mean cutoff was calculated from both cut-off control wells. Normalized absorbance relative to the cutoff was calculated by dividing sample absorbance by mean cutoff control absorbance, then multiplying by 10. Samples with normalized absorbance of 11 or above were considered positive.

### Microneutralization

All sera were heat inactivated (HI) at 56°C for 30 min prior to testing. HI serum was diluted initially 10-fold by combining 10.5 µL HI serum with 94.5 µL DMEM-10, followed by five 3-fold dilutions by serially transferring 35 µL of diluted serum into 70 µL of DMEM-10, resulting in a final dilution series from 10-fold to 2,430-fold and a final volume of 70 µL. These serial dilutions of serum were diluted an additional 2-fold to a final volume of 140 µL with 70 µL of RSV A2, diluted to form a final of 120-250 ffu/well in the assay, resulting in a final dilution series of 20-fold to 4,860-fold. Serum-virus mixtures were incubated at 37°C 5% CO_2_ for 1 h. 96-well plates seeded with 10,000 VeroE6 cells/well were infected with 50 µL/well of serum-virus mixture in duplicate. For each run and sample, results from duplicate measurements were utilized to determine the sample ND50 and ND80 values. Neutralizing titers were based on single run unless otherwise specified. Serial dilutions were added to rows B-G of columns 2-9. Columns 1 and 12 were left empty. Rows A and H of columns 2–11 contained only cells without virus or serum (cell control). Rows B-G of columns 10 and 11 received virus but not serum, serving as virus-only controls. Plates were incubated at 37°C 5% CO_2_ for 26–30 h before fixation. Exogenous complement, typically (20, 62) used to boost neutralizing signal from low-titer sera, was not used in our RSV FRNT; instead, samples with neutralizing titers below the lower limit of quantitation were retested at a lower, fivefold initial dilution to obtain reportable ND50 and ND80 values within assay analytical measurement range (20–4,860).

### Focus-forming assay

Assay plates were fixed for 60 min at room temperature (approximately 21–25°C) with 25 µL (1/2 well volume) of 4% formaldehyde prepared in DPBS with 100 mg/L calcium and 100 mg/L magnesium. After fixation, plates were washed three times with 150 µL room-temperature 1×PBS and incubated for 2 h at room temperature with 50 µL/well of mouse mAb 131-2G against RSV-A2 F-protein (Sigma-Aldrich, MAB8582) diluted 4,000-fold in FFA staining buffer (composed of 1 × PBS, 1 mg/mL saponin, and 0.1% IgG-free BSA). Plates were then washed three times with 150 µL room-temperature FFA wash buffer (composed of 1 × PBS and 0.05% Triton X-100). Plates were incubated for 1 h at room temperature with 50 µL/well of peroxidase-conjugated secondary antibody (Bethyl Laboratories, A90216P) diluted 4,000-fold in FFA staining buffer. Following secondary incubation, plates were then washed 3× with 150 µL room-temperature FFA wash buffer. Plates were developed with 50 µL TrueBlue peroxidase substrate (KPL, 5510-0054) for 15 min at room temperature. Development was stopped by washing three times with 150 µL room-temperature deionized water, and the plates were dried for 10–15 min to minimize visual artifacts from liquid.

### Imaging and counting

Plates were scanned and counted on the S6 Universal M2 ImmunoSpot analyzer (Cellular Technology Ltd, Cleveland, OH). Initial sensitivity/detection settings were calibrated via the Smart Well feature trained on no-serum, virus-only control wells. Counted well area was reduced to 80% without normalization to reduce spurious counting of well shadows. Spot separation was set to 25 to avoid counting larger, single foci as multiple foci. Background balance was set to 0 to reduce counting background artifacts in the form of diffuse, low contrast putative foci. Sample sensitivity was held between 197 and 210 and adjusted to ensure that virus only control well counts numbered 150–250 and that foci counted in no-serum, no virus wells, typically from fibers or plate markings, were <5 foci. Counts were used to determine percent foci reduction at each dilution.

### Data analysis

For each specimen, the response between percent foci reduction and serum dilution (i.e., the effect of serum dilution on neutralizing activity) was modeled via 4-parameter logistic regression (Supplementary Material). The dilutions at which 50% and 80% reduction in neutralizing activity was observed were marked as the ND50 and ND80, respectively. Modeling was performed using the lmfit python package using the Nelder-Mead method. Constraints used were a hill slope between −1.2 and −0.7 (initial guess = −0.8), upper limit between 0.95 and 1.05 (initial guess = 1), lower limit between −0.05 and 0.05 (initial guess = 0), and inflection point between 0 and infinity (initial guess = 50). Code used for analysis can be found here: https://github.com/greninger-lab/rsv_neut_utils. Curve fitting for comparing RSV FRNT ND50 with ELISA absorbance was performed with R package dr4pl (73, 74), using the Nelder-Mead method. Constraints used were a hill slope between 0.3 and 1.2 (initial guess = 0.5), upper limit between 0 and 1,000 (initial guess = 200), lower limit between −100 and 100 (initial guess = −1), and inflection point between negative infinity and 15,000 (initial guess = 10,000). ND50 or ND80 measurements outside the AMR (below 20 or above 4,860) were not used in downstream analysis; samples generating these measurements were flagged to be rerun with a different pre-dilution to obtain measurements within the CMR.

## SUPPLEMENTAL MATERIAL

Figure S1 spectrum.01739-25-s0001.pdf Collection time and titers for specimens

Supplemental tables spectrum.01739-25-s0002.xlsx Tables S1 to S6.

ASM does not own the copyrights to Supplemental Material that may be linked to, or accessed through, an article. The authors have granted ASM a non-exclusive, world-wide license to publish the Supplemental Material files. Please contact the corresponding author directly for reuse.
