# Supplementary material for: Corrected and republished from: “Clinical validation of an RSV neutralization assay and analysis of cross-sectional sera associated with 2021–2023 RSV outbreaks to investigate the immunity debt hypothesis”
Source: Microbiol Spectr. 2025 Dec 23;14(2):e01739-25. doi: 10.1128/spectrum.01739-25 (PMC12889121; doi:10.1128/spectrum.01739-25)
Supplement: Figure S1 — Collection time and titers for specimens [file spectrum.01739-25-s0001.pdf]

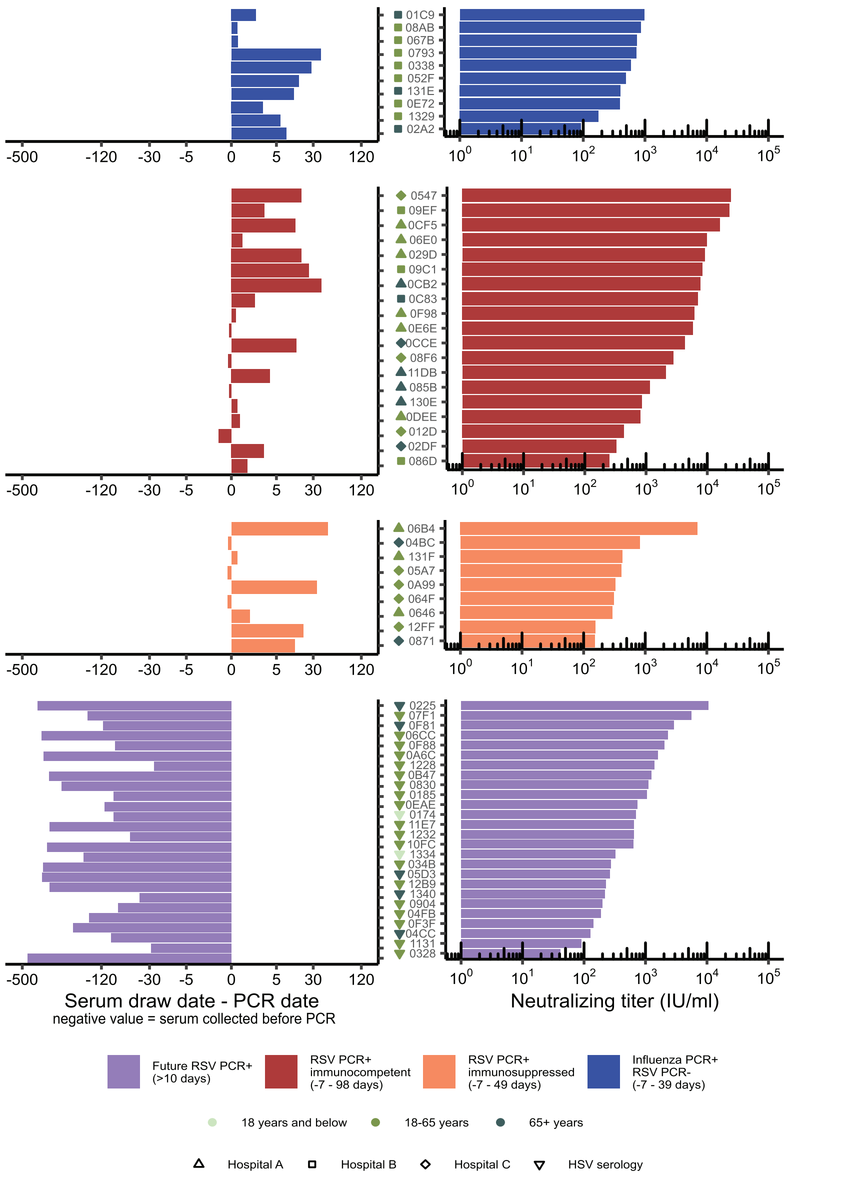

Figure S1: Time delta between serum and RSV PCR test, with neutralizing titers as international units (IU)/mL values. Hospital names were anonymized and patients given random hexadecimal identifiers. Left: Difference in days between serum collection and RSV PCR date, calculated as (serum collection date - PCR test date). Negative values indicate PCR tests taken after serum draw. Right: IU/mL measurements. Influenza PCR+, RSV PCR-: emergency department (ED) patients with no recorded RSV-PCR-positive or immunosuppression status, who tested PCR-positive for influenza from 39 days before to 7 days after serum collection. RSV+, immunocompetent: ED patients PCR-positive for RSV from 98 days before to 7 days after serum collection date. RSV+, immunosuppressed: ED patients PCR-positive for RSV from 49 days before to 7 days after serum collection date, with medical status of immunosuppression at time of serum draw. Future RSV PCR+: individuals with serum drawn 25 days or more before RSV-positive PCR test.
